# Antibody and Antigen Prevalence as Indicators of Ongoing Transmission or Elimination of Visceral Leishmaniasis: A Modeling Study

**DOI:** 10.1093/cid/ciab210

**Published:** 2021-06-14

**Authors:** Luc E Coffeng, Epke A Le Rutte, Johanna Munoz, Emily Adams, Sake J de Vlas

**Affiliations:** 1 Department of Public Health, Erasmus MC, University Medical Center Rotterdam, Rotterdam,The Netherlands; 2 Department of Epidemiology and Public Health, Swiss Tropical and Public Health Institute, Basel,Switzerland; 3 University of Basel, Basel, Switzerland; 4 Department of Tropical Disease Biology, Liverpool School of Tropical Medicine, Liverpool, United Kingdom

**Keywords:** visceral leishmaniasis, control, monitoring, serology, antigenemia

## Abstract

**Background:**

Control of visceral leishmaniasis (VL) on the Indian subcontinent has been highly successful. Control efforts such as indoor residual spraying and active case detection will be scaled down or even halted over the coming years. We explored how after scale-down, potential recurrence of VL cases may be predicted based on population-based surveys of antibody or antigenemia prevalence.

**Methods:**

Using a stochastic age-structured transmission model of VL, we predicted trends in case incidence and biomarker prevalence over time after scaling down control efforts when the target of 3 successive years without VL cases has been achieved. Next, we correlated biomarker prevalence with the occurrence of new VL cases within 10 years of scale-down.

**Results:**

Occurrence of at least 1 new VL case in a population of 10 000 was highly correlated with the seroprevalence and antigenemia prevalence at the moment of scale-down, or 1 or 2 years afterward. Receiver operating characteristic curves indicated that biomarker prevalence in adults provided the most predictive information, and seroprevalence was a more informative predictor of new VL cases than antigenemia prevalence. Thresholds for biomarker prevalence to predict occurrence of new VL cases with high certainty were robust to variation in precontrol endemicity.

**Conclusions:**

The risk of recrudescence of VL after scaling down control efforts can be monitored and mitigated by means of population-based surveys. Our findings highlight that rapid point-of-care diagnostic tools to assess (preferably) seroprevalence or (otherwise) antigenemia in the general population could be a key ingredient of sustainable VL control.

Visceral leishmaniasis (VL), also known as kala-azar, is a vector-borne infection that can lead to long-lasting fevers and death if left untreated, although most infected individuals will remain asymptomatic [1, 2]. In the Indian subcontinent, where the disease is considered to only occur in humans, about 5%–10% of those who are treated for VL develop post–kala-azar dermal leishmaniasis (PKDL), an infectious but self-limiting skin condition [3]. In 2005, India, Nepal, and Bangladesh committed to controlling VL by signing a trilateral memorandum of understanding. The associated continent-wide control measures led to a steep decrease of reported VL incidence from 32 803 in 2005 to 3128 in 2019, representing a 90% drop [4]. Control measures consist of vector control through indoor residual spraying (IRS) of insecticide and active case detection (ACD) followed by prompt treatment, which is provided for free [5]. The goal is to achieve elimination of VL as a public health problem on the Indian subcontinent, which is defined as <1 new or recurring VL case per 10 000 individuals per year at the district or subdistrict level [6]. To validate achievement of the target, the World Health Organization (WHO) requires that the VL incidence in a region be below the target for 3 years in a row in combination with extensive testing. After validation, control measures can be scaled down [7].

Many Indian subdistricts have entered or will be entering this validation phase in the coming years. The prospect of scaling down or even halting control measures without complete interruption of transmission poses a risk of recrudescence of infection. This highlights the need for continued monitoring of ongoing infection. In this study, we assessed the predictive power of population surveys that use serological tests (eg, the direct agglutination test [DAT] for antileishmanial antibody detection) or antigen tests to predict the occurrence of new VL cases (reported or unreported) after scaling down control efforts. To this end, we developed a new stochastic age-structured VL transmission model that is based on an established deterministic VL transmission model [8–11].

## METHODS

### Model Structure and Quantification

We used an established age-structured deterministic VL transmission model that describes the transmission of VL between humans and sand flies on the Indian subcontinent [8–11]. To realistically simulate the probability of interrupting VL transmission, we developed a new stochastic version of the model, considering a finite and discrete number of homogeneously mixing human individuals while keeping the sand fly part of the model deterministic. In the model, most infected individuals remain asymptomatic and recover without ever having symptoms; a small fraction (approximately 1.5%) of individuals become symptomatic and will require treatment or die otherwise; and a small fraction (approximately 3%) of symptomatic cases is assumed to recover spontaneously. Transmission is driven by exposure to sand flies, where sand fly abundance is assumed to peak from July to September of each year [12]. Sand flies can pick up infection from symptomatic cases as well as individuals with PKDL, a self-limiting but long-lasting skin condition that occurs in a fraction of individuals treated for VL (2.5% in the model [3]). In addition, we distinguish the possibility that asymptomatically infected individuals do (model E1) or do not (model E0) contribute to transmission. The model incorporates IRS coverage through a proportional reduction in the sand fly population density and ACD through a decrease in the average detection delay of symptomatic cases (precontrol delay of 60 days [9, 13]). In the model, we assume that seropositivity based on the DAT is associated with the late asymptomatic stage of infection (ie, after individuals have been infected for a while) as well as the symptomatic stages and the early recovered stage (after which DAT positivity, and thus the humoral immune response, is lost again) [8]. DAT positivity was defined at the 1:800 titer cutoff, instead of the standard of 1:1600, which increases test sensitivity but decreases specificity. In contrast, we assume that antigenemia (ie, the persistence of antigen in circulating blood) is only associated with the late asymptomatic stage and the symptomatic stages when parasite loads are presumably highest. The model assumes that no infection can be introduced from outside the population.

Model parameters were previously calibrated based on age-structured data from approximately 21 000 individuals included in the KalaNet bednet trial in India and Nepal [8, 14, 15]. Age patterns in DAT and polymerase chain reaction positivity (where polymerase chain reaction positivity included the stage of infection that we consider antigen positive, along with the early stage of asymptomatic infection) were reproduced assuming that exposure to bites of sand flies increase linearly from zero at birth to a maximum value at age 20 years, and plateaus thereafter (crudely reflecting change of body surface area with age). The impact of IRS was previously estimated using a geographical cross-validation on decreasing case incidence in Bihar (approximately 6000 VL cases in 8 districts over a period of 18 months) [9, 13]. A schematic representation of the model structure and an overview of model parameter values are presented in the Supplementary Figure 1 and Supplementary Table 1, respectively.

The model was coded in R (version 4.0.2), using the *pomp* package (version 3.1.1.7); the model code is publicly accessible at https://gitlab.com/erasmusmc-public-health/vl-serosurveys. The Policy-Relevant Items for Reporting Models in Epidemiology of Neglected Tropical Diseases Summary is provided in Supplementary Table 2, which was previously established to set a standard and increase consistency among modeling studies that aim to inform policy [16].

### Simulation Scenarios

With each version of the stochastic transmission model (E0 and E1), we performed 10 000 repeated simulations for a stable population of 10 000 people. Transmission conditions (ie, the sand fly to human ratio) were allowed to vary randomly between simulations such that the precontrol VL incidence ranged from 2 to 15 reported VL cases per 10 000 capita per year, as expected under the deterministic version of the model. During the precontrol phase, we assumed that only passive case detection was in place, leading to an average duration between the start of symptoms and the start of treatment of 60 days [13]. To start a stochastic simulation, we extracted the expected precontrol equilibrium state of the human and fly population (ie, distribution over model compartments) from a deterministic simulation based on the same transmission conditions. This population state was used to seed 10 000 individuals across age and disease compartments via a draw from a multinomial distribution. Based on this seeding, we ran the stochastic model, implementing control measures as recommended by WHO until zero VL cases were observed (ie, only reported cases) for 3 consecutive calendar years (ie, a period starting on 1 January and ending 31 December, 3 years later). Control efforts were assumed to start with a 5-year attack phase in which we assumed that IRS covers 67% of the population and that ACD reduces the time to treatment to an average of 45 days. Subsequently, during the so-called consolidation phase, IRS coverage was reduced to 45%, but ACD efforts are further increased leading to an average duration to treatment of 30 days. Achievement of the target (3 consecutive years with zero reported VL cases, with rigorous ACD) was assessed from completion of the attack phase onward, meaning that at least 8 years of control had been performed before any control program was allowed to scale down. If a simulation did not achieve the target of 3 consecutive years with zero reported VL cases within 20 years of control efforts (which happens more often if asymptomatic infections do not contribute to transmission [9], ie, in model E0), it was discarded and excluded from further analysis. For those simulations in which the target was met, control efforts were scaled down to the precontrol situation (no IRS and no ACD such that the average detection delay returned to 60 days) and the simulation was continued for another 10 years to see if any new VL cases (reported or unreported) would occur.

To see if the occurrence of at least 1 new VL case could be predicted based on prevalence of biomarkers in the population, we saved model-predicted trends in age-specific prevalence of DAT and antigenemia prevalence. Receiver operating characteristic (ROC) curves as well as positive and negative predictive values (PPV and NPV) of thresholds for biomarker prevalence were calculated for 3 time points: at the moment of scaling down control efforts, and 1 and 2 years after the scale-down. For the last 2 time points, we excluded simulations in which 1 or more VL cases were already detected before the time of the survey, assuming that this observation alone would already lead to policy action. For each survey, we assumed that a random subset of 500 individuals in the population is tested, simulating 100 random surveys for each time point in each simulation. Simulated surveys sampled either only preschool-age children (aged 0–4 years), school-age children (aged 5–14 years), or adults (aged ≥15 years). Threshold values were expressed in terms of the number of biomarker-positive individuals among this sample of 500 at or above which we expect recurrence of at least 1 VL case in the 10 years following scaling down of control efforts.

## RESULTS

Of the 10 000 repeated stochastic simulations that were performed with each model variant, in 8011 (model E1) and 4705 simulations (model E0) the target of 3 consecutive years of zero reported VL cases was achieved within 20 years of control, and control could be scaled down to passive case detection only. The probability of at least 1 new VL case occurring within 10 years after scaling down control was higher in model E0 (2936 of 4705 = 62.4%) than in model E1 (3028 of 8011 = 37.8%). If 1 or more new VL cases occurred, the first case typically occurred within the first year after scaling down (96.5% of simulations in model E1 and 69.3% in model E0) but with a very long right tail (up to 5 years for model E1 and 10 years for model E0) (Supplementary Figure 2). The timing of the first detected case was slightly later, although still typically within 1 year after scaling down (93.7% of simulations in model E1 and 60.3% in model E0).

**Figure 1.**
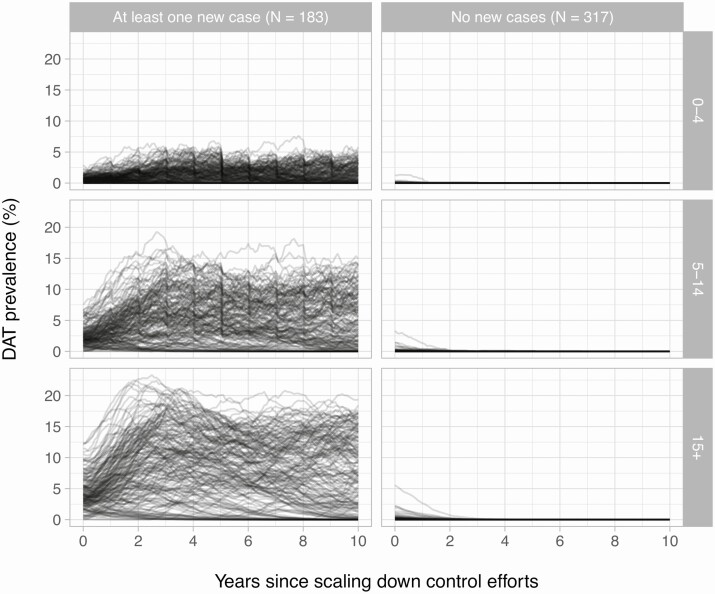
Model-predicted trends in age-specific DAT prevalence after scaling down control efforts against visceral leishmaniasis (VL). Lines represent biomarker prevalence from a randomly selected subset of 500 simulations. Rows represent different age categories; columns depict simulations that resulted in occurrence (left) or absence of new VL cases (right), with the total number of simulations per outcome indicated at the top of each column (N). Predictions are based on the assumptions that both symptomatic and asymptomatic infections contribute to transmission (model E1) and that all individuals are tested. Similar predictions that assume asymptomatic infections do not contribute to transmission (model E0) can be found in Supplementary Figure 4. Abbreviation: DAT, direct agglutination test.

If a new VL case occurred after scaling down control, model E1 predicted that DAT prevalences were above 0% for at least 2 years after scale-down (100% of simulations; Supplementary Table 3) and fluctuated and rose over time (Figure 1). DAT prevalences reached higher values in adults (age 15+) than in preschool (age 0–4) and school-age children (age 5–14), reflecting the assumed age patterns in exposure to sand fly bites. If no new VL cases occurred and DAT prevalences were above 0% at the moment of scale-down (45% of the simulations), the DAT prevalence quickly declined to values close to zero within 1 to 2 years. Trends in antigenemia prevalence followed a similar pattern, although with overall lower prevalences compared with DAT (Supplementary Figure 3), reflecting that the duration of antigen-positivity is shorter than that of seropositivity. If no new VL case occurred after scale-down, antigenemia prevalence was rarely above 0% at the moment of scale-down (13% of simulations); if a new VL case did occur, antigenemia prevalence was almost always above 0% (99% of simulations; Supplementary Table 4). Predicted trends in biomarker prevalence and the differences in trends between simulation outcomes (absence vs occurrence of new VL cases) were qualitatively similar between model E1 (Figure 1 and Supplementary Figure 3) and model E0 (Supplementary Figures 4 and 5). DAT prevalences in children aged <15 years were slightly lower at the turn of each year due to the fact that at that moment in the simulation, for efficiency, all individuals were assumed to simultaneously age by 1 year. This modeling artifact is not visible for antigenemia prevalence due to the higher level of stochastic noise associated with overall lower prevalences.

**Figure 2.**
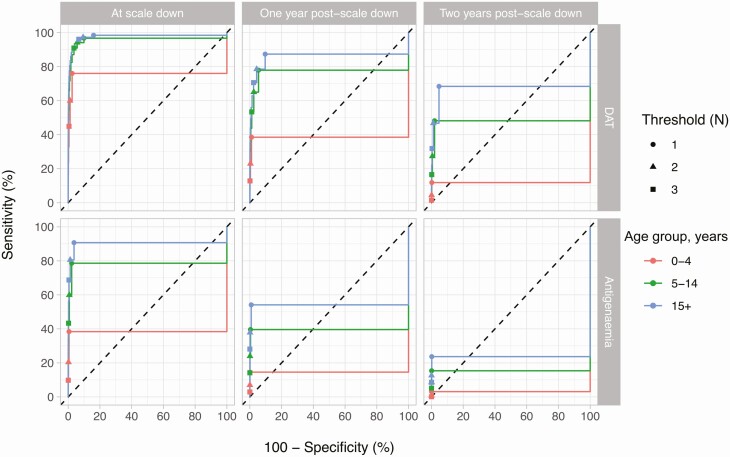
Receiver operating characteristic (ROC) curve for predicting occurrence of new VL cases based on age-specific prevalence of direct agglutination test or antigenemia measured at and up to 2 years after scaling down control efforts. Columns depict ROC based on biomarkers measured at 3 time points; rows depict different biomarkers. Symbols indicate thresholds for the number (N) of biomarker-positive cases at or above which the recurrence of at least 1 visceral leishmaniasis case was predicted. Predictions are based on the assumptions that both symptomatic and asymptomatic infections contribute to transmission (model E1) and that 500 individuals are tested for biomarker positivity. Similar predictions that assume asymptomatic infections do not contribute to transmission (model E0) can be found in Supplementary Figure 6.

Sensitivity and specificity of different thresholds for biomarker prevalence as predicted by model E1 for samples of 500 individuals are illustrated as ROC curves in Figure 2. Because biomarker prevalences were generally higher in adults (age 15+) and thus suffered less from stochastic noise, sensitivity and specificity of thresholds were highest when applied to prevalences in that particular age group. This pattern was qualitatively similar for model E0 (Supplementary Figure 6), although sensitivity and specificity were lower overall than in model E1 and the difference in ROC curves based on biomarker prevalences in adults (15+ years) and school-age children (5–14 years) was somewhat larger. Still, the 2 model variants agreed that a threshold of 1 to 3 biomarker-positive cases in a sample of 500 was optimal to achieve both high sensitivity and specificity and that DAT prevalence was a more informative predictor of recurrence of VL cases than antigenemia prevalence. Furthermore, the model variants agreed that when biomarker prevalence was measured 1 or 2 years after scaling down control (excluding simulations in which a new VL case had already been detected before that time), sensitivity decreased strongly and specificity increased slightly.

**Figure 3.**
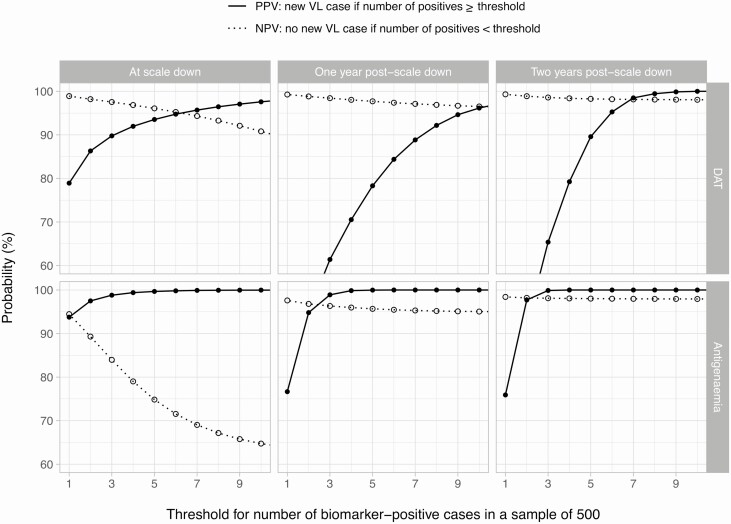
PPV and NPV of direct agglutination test and antigenemia prevalence in adults (age 15+ years) measured up to 2 years after scaling down control efforts, given a choice of threshold value. Columns depict curves based on biomarkers measured at 3 time points; rows depict different biomarkers. Note that the predictive values based on biomarker prevalences measured 1 or 2 years after scale-down (middle and right panels) are conditional on no new VL cases having been detected since scale-down. Predictions are based on the assumptions that both symptomatic and asymptomatic infections contribute to transmission (model E1) and that 500 individuals are tested for biomarker positivity. Similar predictions that assume asymptomatic infections do not contribute to transmission (model E0) can be found in Supplementary Figure 7. Abbreviations: NPV, negative predictive value; PPV, positive predictive value; VL, visceral leishmaniasis.

The PPV and NPV of thresholds for biomarker prevalence, as predicted by model E1, depended on both the biomarker as well as the time of measurement (Figure 3). In general, NPVs (ie, the probability of no more VL cases if the biomarker prevalence was under the threshold) were higher for DAT (up to 99%) than for antigenemia prevalence (up to 95%), as the latter were limited by the fact that the prevalence of antigen positivity is relatively low compared with DAT prevalence. For DAT prevalence, a threshold value of 6 positive individuals in a sample of 500 corresponded to a ≥95% probability that no further VL cases could occur if DAT prevalence was measured at the moment of scale-down. For antigenemia, this NPV of 95% could only just be achieved by a threshold of <1 positive individual. The corresponding PPVs (ie, the probability of recurring VL cases if biomarker prevalence is above or equal to the threshold) were ≥94% for both biomarkers. For both biomarkers, when prevalence was measured 1 or 2 years after scale-down, the PPV decreased and the NPV increased and flattened as a function of threshold value (ie, if no new VL case had been detected by then, the a priori probability of recurrence had already strongly declined). Alternatively, when assuming that asymptomatic infections do not contribute to transmission (model E0), in general, NPVs were lower for DAT (80% to 90% for a threshold of 1 case) and particularly lower for antigenemia (60% to 70% for a threshold of 1 case), although PPVs were still high (≥90%) (Supplementary Figure 7). In contrast to model E1, in model E0, measuring biomarker prevalence 1 or 2 years after scaling down only marginally affected the PPV yet still increased the NPV. Last, PPVs were robust to variation in both precontrol endemicity models E1 and E0 (Supplementary Figures 8 and 9), provided that the biomarker and associated threshold prevalence are chosen aiming at a PPV and NPV ≥90%. Overall, NPVs were slightly higher for settings with high precontrol endemicity, reflecting that achieving a prevalence under the threshold despite more intense transmission conditions was highly indicative of successful control.

## Discussion

We show that occurrence of new VL cases (reported or unreported) after achieving 3 years of zero reported VL cases and scaling down control efforts is highly correlated with the seroprevalence (based on DAT) and antigenemia prevalence. Biomarker prevalence in adults (aged ≥15 years) provides the most predictive information on prospects of resurgence, and DAT prevalence appears to be a more informative predictor than antigenemia prevalence. Thresholds for biomarker prevalence to predict occurrence of new VL cases with high certainty were robust to variation in precontrol endemicity, allowing thresholds to be applied without exact knowledge of transmission conditions.

For an area covering a population of 10 000 people, having no DAT-positive individuals in a sample of 500 (ie, a threshold of <1 case) at the moment of scale-down is a conservative approach to predicting whether or not new VL cases will occur. However, the predictive power of such a criterion does depend on whether or not asymptomatic individuals do (NPV ≥99%, PPV ≥79%) or do not contribute to transmission (NPV ≥80%, PPV ≥92%). In general, the predictive value could be further boosted by sampling more individuals (and adapting the threshold criterion accordingly) or by performing repeated surveys over time (eg, annually). Of course, as this is a modeling study and given that no monitoring program exists as suggested here, predictions should be validated based on field data, ideally longitudinal data of biomarker prevalence from areas that have implemented the control measures recommended by the WHO.

A major strength of this study is that the calculated threshold values for the number of biomarker-positive cases in a sample of 500 individuals can be readily used for interpretation of field data (based on the same sample size) without the need for calculating confidence bounds around prevalence estimates. This is possible because stochastic as well as statistical uncertainty are captured in our calculations: stochastic uncertainty via many repeated simulations of the population dynamics over time, and statistical uncertainty via 100 repeated survey simulations within each transmission simulation. In addition, the methodology that we used can be easily adapted to other population sizes and survey sample sizes. However, for much larger population sizes, model predictions should ideally be based on a metapopulation model that accounts for the fact that individuals live in subpopulations and do not mix homogeneously. Such a model would also address the most important limitation of this study that we assume a closed population without the possibility of introduction of infection from outside. Recrudescence of VL transmission through introduction via human mobility is a real risk, unless the strategy is successfully rolled out on a very large scale. As such, the threshold for biomarker prevalence proposed here should be interpreted as an indicator of recurrence of VL transmission through local transmission. The metapopulation model that is needed to relax this assumption is currently being developed.

Our finding that thresholds for biomarker prevalence were quite robust to variation in precontrol endemicity is extremely convenient for policy development and implementation of monitoring in field settings. This robustness is in stark contrast with, for example, thresholds for Ov16 seroprevalence to evaluate progress toward elimination of onchocerciasis, for which threshold values for a particular target predictive value strongly change with precontrol endemicity [17]. This is not the case for VL because of the precondition of 3 consecutive years of zero cases before scaling down control efforts. This ensures that if the target is met despite a high transmission potential, it is indicative of having interrupted transmission with high probability. Of course, this is conditional on meeting the target through high detection effort rather than low detection effort (causing fewer cases to be reported), for which we previously showed that it can lead to a “confirmatory” drop in case rates while true case numbers are rising [18]. Then again, if the monitoring strategy developed in this study would be applied to a situation where the target was met due to poor case detection, we expect that this would most likely be immediately reflected by a high biomarker prevalence.

According to our findings, measuring biomarker prevalence 1 or 2 years after scaling down control efforts (as long as no new VL cases have been detected in the meanwhile) increases the predictive power for absence of new VL cases (ie, NPV), which was already very high when biomarker (especially DAT) prevalence was assessed at the moment of scale-down. This finding was mostly driven by the fact that if VL transmission resurged, it did so mostly within 1 year after scale-down. In other words, if no new VL cases were reported in the first year after scale-down, the probability of resurgence was very low to begin with, which drove up the NPV (and lowered the PPV). It is therefore important to note that the benefit of this higher NPV comes at the cost of delayed information, giving the infection more opportunity to be transmitted further throughout the population in case of resurgence. Given that in case of VL recurrence, this typically happened within 1 year of scaling down, we recommend that a biomarker survey be performed at the moment of scale-down or slightly before. However, if the biomarker survey is performed much earlier than the 3-year benchmark (say, 1 year before), threshold values for the number of positive cases would have to be adapted and might even depend more on precontrol endemicity.

It is important to note that for the sake of illustrating the potential predictive value of biomarkers, in our simulations, we assumed that diagnostic tests for antibodies or antigens are 100% sensitive and specific at the individual level. However, in reality, false-positive and false-negative results should certainly be expected to occur, adding noise to observations and lowering the PPV and NPV of biomarker prevalence for recurrence of VL cases compared with what we estimate here. Also, it should be noted that our model predictions for DAT prevalence are quantified based on the diagnostic techniques and titer thresholds used to calibrate our model to data from the KalaNet study [14, 15]. For a more sensitive monitoring strategy (ie, a higher chance of correctly identifying settings where new VL cases will occur), it might be advantageous to use a lower titer threshold for DAT positivity, although this would come at a cost of lower specificity (ie, a higher risk of continuing control efforts longer than strictly necessary). An added benefit would be that the use of a lower titer threshold would result in higher prevalences overall, which would allow for a higher threshold of DAT prevalence in the survey sample and thus requiring a smaller sample size to conclude that the prevalence is statistically significant under the threshold. It is possible to capture this in our model but requires further model development and calibration using more detailed data on actual DAT titers. Data from the follow-up KalaNet study [19] will be particularly valuable here, as it would also allow us to further validate model predictions over time. Antigenemia, on the other hand, was not found to be as good a predictive marker as DAT. This is presumably because individuals have a high detectable antigen load for a short period of time, especially when compared with the duration of detectable antibodies, and therefore the cycle of surveillance may not capture positive cases, especially with low numbers of participants surveyed. Antigen tests could instead be used to confirm findings of these population surveys as a highly specific test.

Our findings highlight the need for further operational research into effective monitoring to ensure sustained control of VL on the Indian subcontinent. First, given our finding that population-based surveys can be an extremely valuable monitoring tool when scaling down control, the questions arise, what geographical units such surveys should cover and how should surveys be implemented? For instance, how many communities and how many individuals should be selected per community? This would depend on the expected level of geographical clustering of VL transmission after prolonged control, which needs to be informed by field studies such as the recent KalaNet follow-up study [19]. Second, how feasible is it to sample, say, 500 individuals per 10 000 population? Sampling 5% of the population may be practically too demanding or too expensive, especially when surveys have to rely on laboratory facilities. Therefore, there is an urgent need for rapid point-of-care diagnostic tools to assess seroprevalence or antigenemia in the general population. Of course, to define useful threshold values for biomarker prevalence measured by such tools, their sensitivity and specificity need to be carefully quantified. Third, there is a need to define more specific policy actions for when biomarker surveys indicate ongoing VL transmission while no VL cases have been detected yet. These actions may depend on the encountered prevalence of biomarker positivity. If biomarker positivity is highly clustered, more in-depth follow-up surveys of the locations with high biomarker prevalence may be an adequate first step. If biomarker positivity is more homogeneously distributed over a larger area, more immediate action in terms of reinstating IRS and/or ACD across the entire geographical area may be more appropriate.

We conclude that the risk of recrudescence of VL after scaling down control efforts can be monitored and mitigated by means of population-based surveys. We recommend that such surveys be based on biomarkers of current and recent infection, such as antibodies, as prevalences for such biomarkers are higher than antigen prevalences and thus provide more statistical information. Our findings highlight that rapid point-of-care diagnostic tools to assess (preferably) seroprevalence or (otherwise) antigenemia in the general population could be a key ingredient of sustainable VL control.

## Supplementary Data

Supplementary materials are available at *Clinical Infectious Diseases* online. Consisting of data provided by the authors to benefit the reader, the posted materials are not copyedited and are the sole responsibility of the authors, so questions or comments should be addressed to the corresponding author.

ciab210_suppl_Supplementary-Material

## Notes

***Author contributions.*** L. E. C. conceived the study. J. M. and L. E. C. programmed the stochastic model, ran the simulations, performed the analyses, and drafted the figures. L. E. C., E. A. L. R., E. R. A., and S. J. dV. interpreted the results. L. E. C. and E. A. L. R. drafted the manuscript. All authors critically revised the manuscript for intellectual content and read and approved the final manuscript.

***Disclaimer.*** The views, opinions, assumptions, or any other information set out in this article are solely those of the authors and should not be attributed to the funders or any other person connected with the funders.

***Supplement sponsorship.*** This article appears as part of the supplement “Sustainable control of neglected tropical diseases – surveillance and diagnostics”, sponsored by the NTD Modelling Consortium.

***Financial support.*** This work was supported by the Neglected Tropical Disease NTD Modelling Consortium by the Bill and Melinda Gates Foundation (OPP1184344). L. E. C. further acknowledges funding from the Dutch Research Council (NWO, grant 016.Veni.178.023).

***Potential conflicts of interest.*** All authors: No reported conflicts of interest. All authors have submitted the ICMJE Form for Disclosure of Potential Conflicts of Interest. Conflicts that the editors consider relevant to the content of the manuscript have been disclosed.
