# Supplementary material for: Antibody and Antigen Prevalence as Indicators of Ongoing Transmission or Elimination of Visceral Leishmaniasis: A Modeling Study
Source: Clin Infect Dis. 2021 Jun 14;72(Suppl 3):S180–7. doi: 10.1093/cid/ciab210 (PMC8201595; doi:10.1093/cid/ciab210)
Supplement: ciab210_suppl_Supplementary-Material [file ciab210_suppl_supplementary-material.pdf]

Supplementary Information to "Antibody and antigen prevalence as indicators of ongoing transmission or elimination of visceral leishmaniasis: a modelling study"

Luc E. Coffeng,[a,#] Epke A. Le Rutte,[a,b,c] Johanna Munoz,[a] Emily Adams,[d] Sake J. de Vlas[a]

[a] Department of Public Health, Erasmus MC, University Medical Center Rotterdam, Rotterdam, The Netherlands

[b] Department of Epidemiology and Public Health, Swiss Tropical and Public Health Institute, Basel, Switzerland

[c] University of Basel, Basel, Switzerland

[d] Department of Tropical Disease Biology, Liverpool School of Tropical Medicine, Liverpool, United Kingdom

[#] Corresponding author: Luc E. Coffeng, Department of Public Health, Erasmus MC, University Medical Center Rotterdam, P.O. box 2040, 3000 CA Rotterdam, The Netherlands; l.coffeng@erasmusmc.nl.

Table of contents

Model structure **2**

Model parameters **3**

PRIME-NTD Summary Table **4**

Additional results **5**

References **14**

Model structure

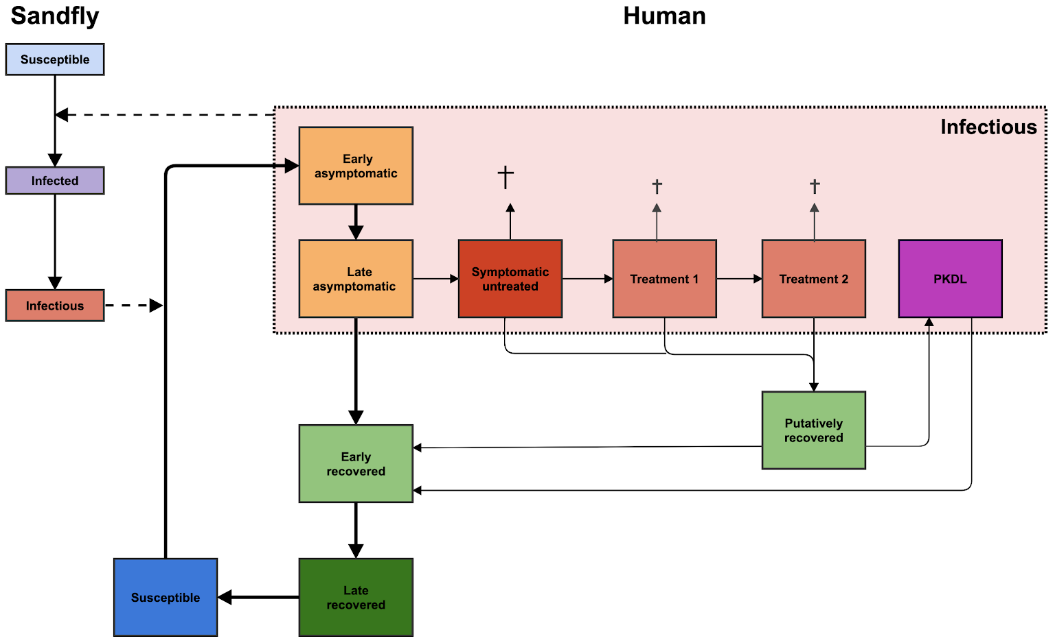

Figure S1. Schematic presentation of the model structure. In both model variants E0 and E1, symptomatic individuals – VL (red) and PKDL (purple) – contribute to transmission. In addition, in model E1, asymptomatic individuals (yellow) contribute to transmission. In model E0, asymptomatic individuals do not contribute to transmission. For the original calibration of the mode, all asymptomatically infected (yellow) and symptomatic stages of infection (red and purple) were considered PCR-positive. DAT-positivity was linked to only the late asymptomatic stage, symptomatic stages, and the putatively and early recovered stages (light green), which we adopted in the current study. In addition, for the current study we consider the late asymptomatic and all symptomatic stages to be antigen positive, assuming that antigen levels will be too low to detect in the early stages of infection.

Model parameters

Table S1. Parameters values used in simulations.

Model parameter	Value[a]	Source
Human birth rate (per 1000 capita)	21 (Indian crude birth rate in 2011)	[1]
Human mortality rate	Age-dependent (Indian mortality rates in 2011)	[2]
Average duration of early asymptomatic stage (days)	382	Fitted to KalaNet data [3,4]
Average duration of late asymptomatic stage (days)	136	Fitted to KalaNet data [3,4]
Average duration of symptomatic untreated stage (days)	60 (pre-control), 45 (attack-phase), 30 (consolidation phase)	[4–6]
Average duration of symptomatic treatment 1 (days)	2.5	[7]
Average duration of symptomatic treatment 2 (days)	10	[4,5,8]
Average duration of putatively recovered stage (months)	21	[9–11]
Average duration of PKDL (years)	5	Expert opinion and [10]
Average duration of early recovered stage	482	Fitted to data in [3,4]
Average duration of late recovered stage (years)	2	Assumption based on [3]
Relative infectiveness of early asymptomatic individuals	0.0144 (model E1) or 0 (model E0)	Fitted to data (E1) [4,12] or pre-set (E0)
Relative infectiveness of late asymptomatic individuals	0.0288 (model E1) or 0 (model E0)	Fitted to data (E1) [3,4] or pre-set (E0)
Relative infectiveness of symptomatic untreated cases	1	Reference value
Relative infectiveness of patients under treatment 1 and 2	0.5	Expert opinion and [3]
Relative infectiveness of PKDL cases	0.9	[13,14]
Fraction of late asymptomatic individuals that become symptomatic untreated	1.4%	Fitted in [3,4]
Fraction of untreated symptomatic cases that spontaneously, putatively recover	0.03	[15]
Excess mortality rate among untreated symptomatic cases (per day)	1/150	Assumption
Excess mortality rate among treated symptomatic cases (per day)	1/120	Assumption [7,8]
Fraction of failed first-line treatments	0.05	Based on data presented in Supplementary File 2 of [4]
Fraction of putatively recovered cases that develop PKDL	0.05	[4,16,17]
Average life expectancy of the sand fly (days)	14	[18,19]
Average duration of incubation period in sandflies (days)	5	[20]
Sand fly biting rate (per day)	0.25	[21,22]
Transmission probability sand fly to human	1.0[b]	Reference value
Age-dependent exposure to sand fly bites (relative to the exposure of an adult person)	Zero at birth and increasing linearly to 1.0 at age 20 and stable from then onwards	Assumption

[a] The parameter values listed here are the same for Models E0 and E1, unless indicated otherwise.

[b] The probability that a susceptible person becomes infected when bitten by an infectious sand fly is assumed to be 1; potential overestimation is compensated by the parameter for sand fly density per human.

PRIME-NTD Summary Table

Table S1. Policy-Relevant Items for Reporting Models in Epidemiology of Neglected Tropical Diseases (PRIME-NTD) Summary Table [23].

**Principle**	**What has been done to satisfy the principle?**	**Where in the manuscript is this described?**
1. Stakeholder engagement	At the end of October 2020, the design of the study and preliminary results were communicated to the WHO NTD office as part of an open online consultation for input on the monitoring and evaluation framework for the 2030 WHO NTD Roadmap	It is not
2. Complete model documentation	Described in detail in previous open access publications and on Github	Referred to previous papers in Methods, link to full open access of model code and documentation on Github in the methods section
3. Complete description of data used	Described in detail in previous publications	Referred to particular datasets and previous papers in Methods [4,12]
4. Communicating uncertainty	Described in detail in previous publications and also highlighted in this paper	Methods [4] / discussion
5. Testable model outcomes	Not yet; in the future the model predictions can be compared to the KalaNet Revisited data	Discussion

Additional results

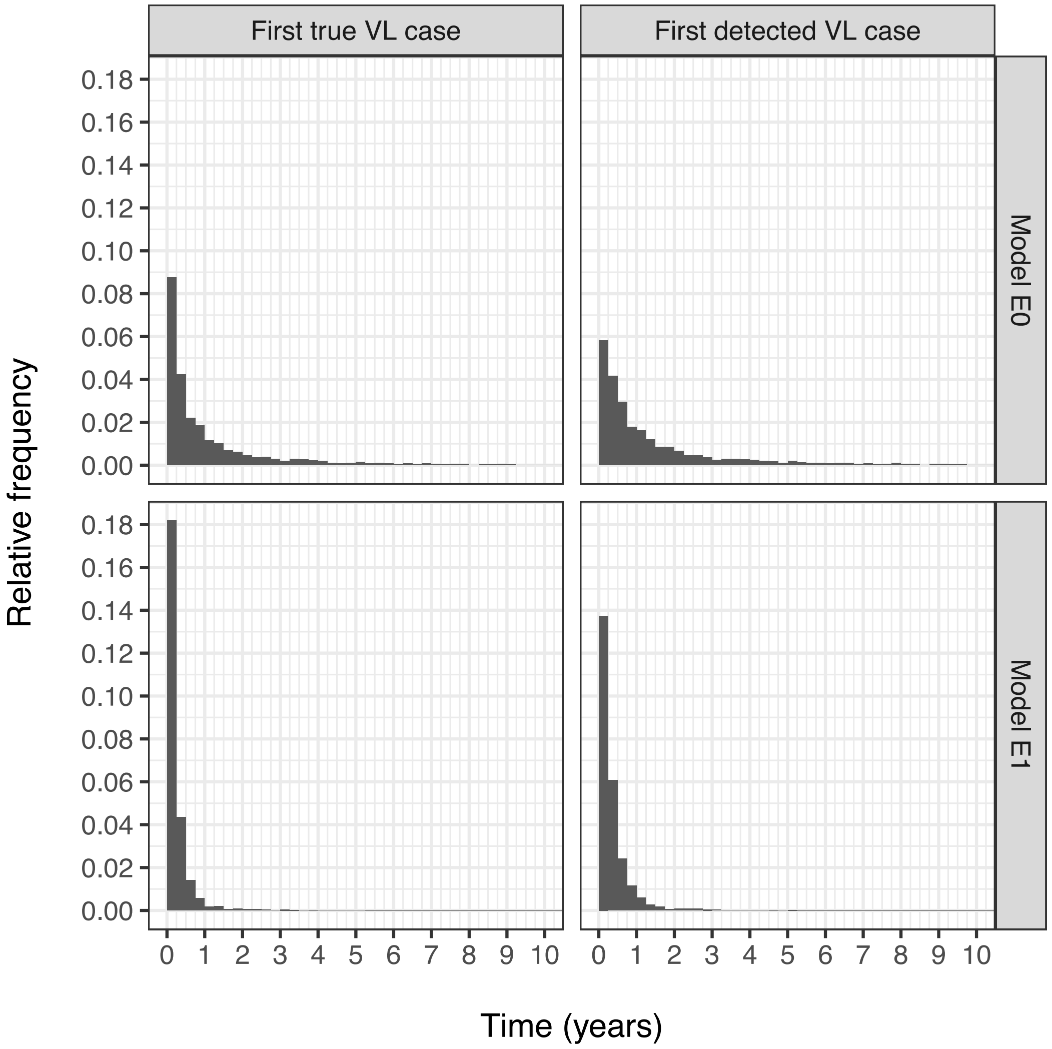

Figure S2. Model-predicted time of occurrence of the first new VL case after scaling down control efforts against visceral leishmaniasis. Histograms show the frequency distribution of time over repeated simulations, for those simulations in which at least one new VL case occurred. The panels on the left show the timing of onset of symptoms of the first VL case, regardless of whether or not that case was detected (bars add up to 1.0). The panels on the right show the timing of when a new VL case was detected for the first time (bars add up to less than 1.0 because in 1%-2% of the simulations the new cases remained undetected).

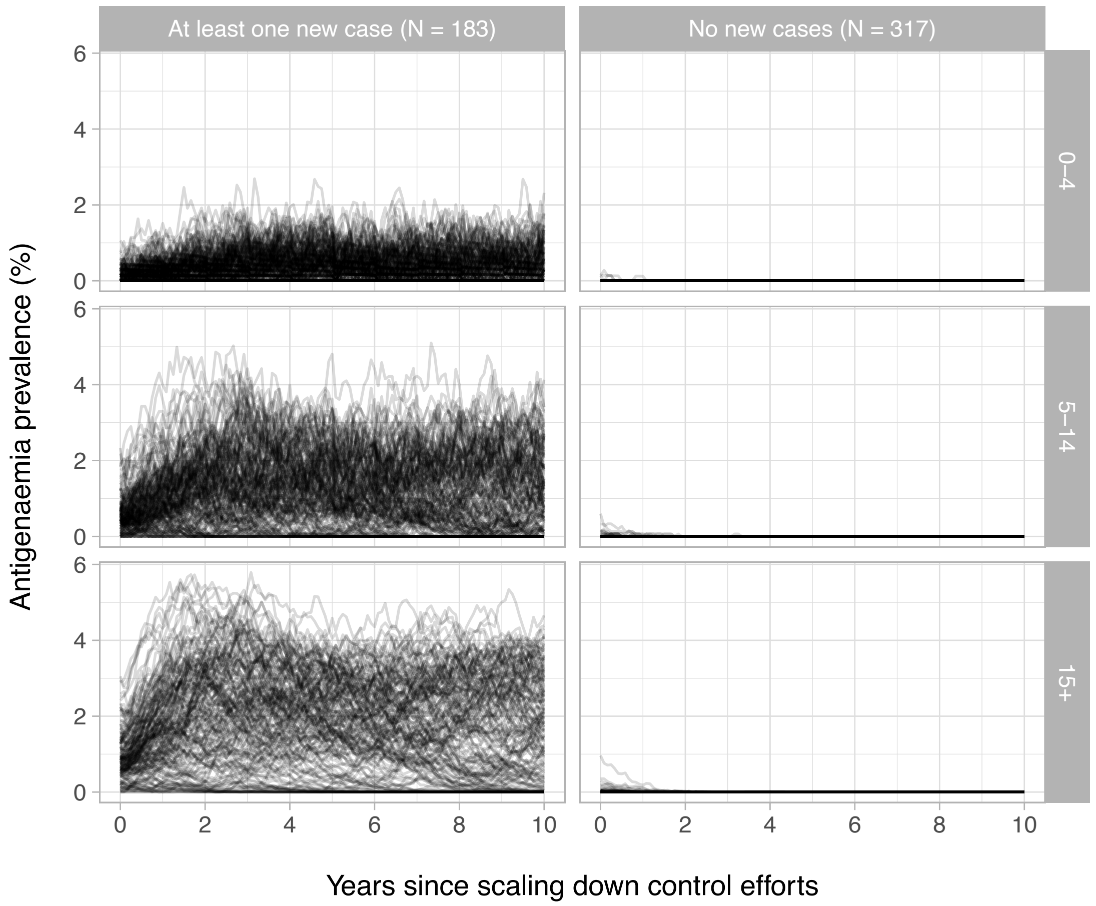

Figure S3. Model-predicted trends in age-specific antigenaemia prevalence after scaling down control efforts against visceral leishmaniasis. Lines represent biomarker prevalence from a randomly selected subset of 500 simulations. Rows represent different age categories; columns depict simulations that resulted in occurrence (left) or absence of new VL cases (right), with the total number of simulations per outcome indicated at the top of each column (N). Predictions are based on the assumption that both symptomatic and asymptomatic infections contribute to transmission (model E1) and that all individuals are tested. Similar predictions assuming asymptomatic infections do not contribute to transmission (model E0) can be found in Figure S5.

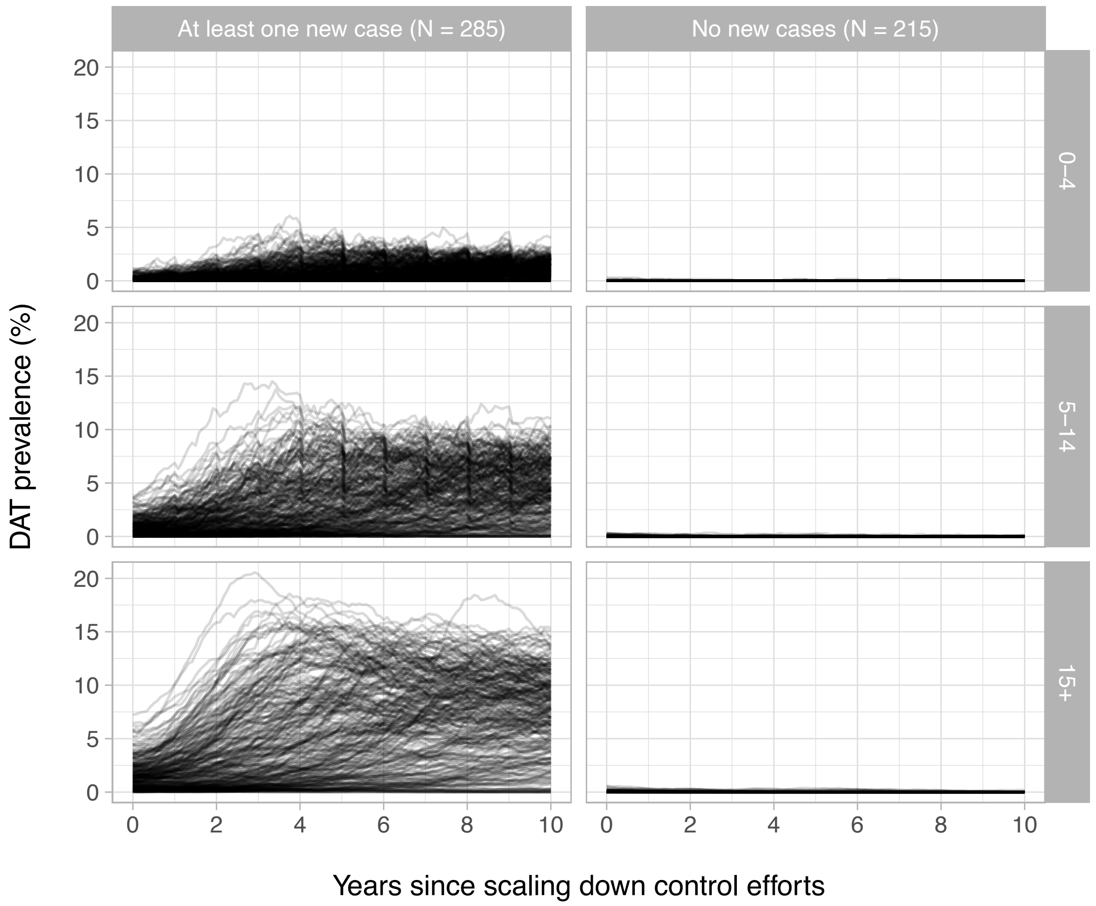

Figure S4. Model-predicted trends in age-specific DAT prevalence after scaling down control efforts against visceral leishmaniasis. Lines represent biomarker prevalence from a randomly selected subset of 500 simulations. Rows represent different age categories; columns depict simulations that resulted in occurrence (left) or absence of new VL cases (right), with the total number of simulations per outcome indicated at the top of each column (N). Predictions are based on the assumptions that asymptomatic infections do not contribute to transmission (model E0) and that all individuals are tested.

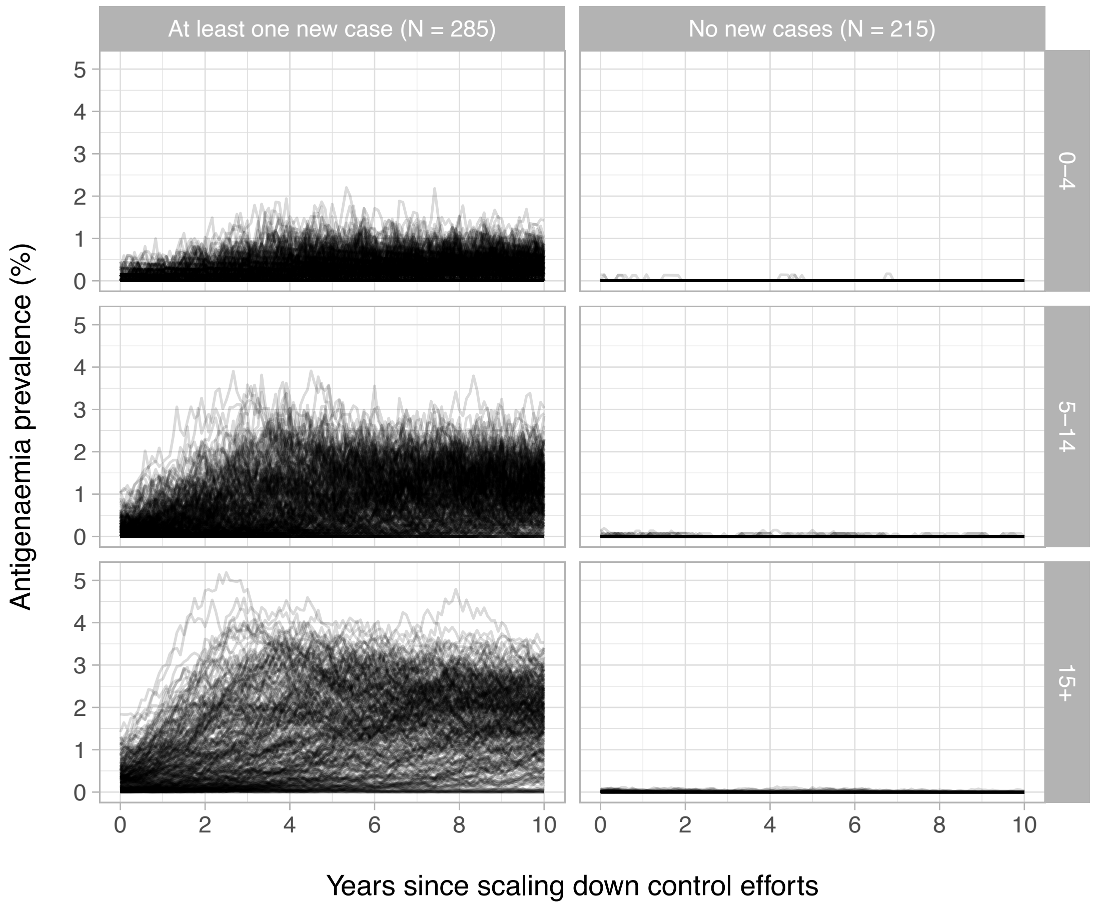

Figure S5. Model-predicted trends in age-specific antigenaemia prevalence after scaling down control efforts against visceral leishmaniasis. Lines represent biomarker prevalence from a randomly selected subset of 500 simulations. Rows represent different age categories; columns depict simulations that resulted in occurrence (left) or absence of new VL cases (right), with the total number of simulations per outcome indicated at the top of each column (N). Predictions are based on the assumptions that asymptomatic infections do not contribute to transmission (model E0) and that all individuals are tested.

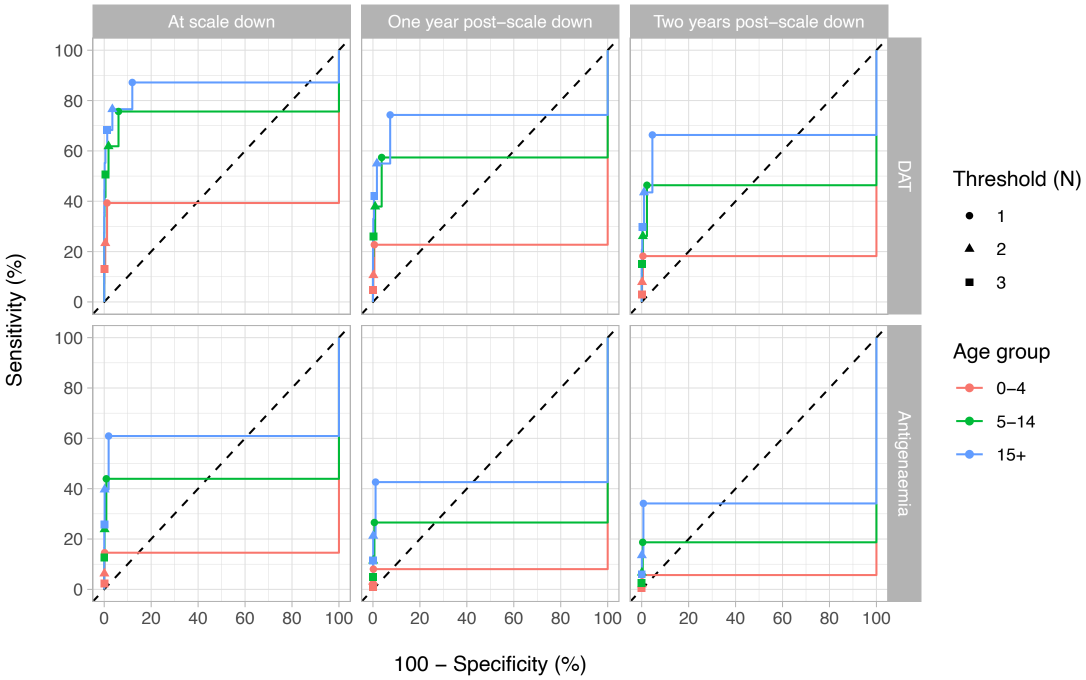

Figure S6. Receiver-operator curve for prediction of recrudescence of transmission based on age-specific prevalence of DAT or antigenaemia up to two years after scaling down control efforts. Rows show receiver-operator curves (ROC) for the two different biomarkers (DAT and antigenaemia prevalence) used to predict occurrence of a new VL case. Symbols indicate thresholds for biomarker prevalence at or above which the recurrence of at least one VL case was predicted. Columns depict ROC curves based on biomarkers measured on three different time points; rows depict different biomarkers. Predictions are based on the assumptions that asymptomatic infections do not contribute to transmission (model E0) and that 500 individuals are tested for biomarker positivity.

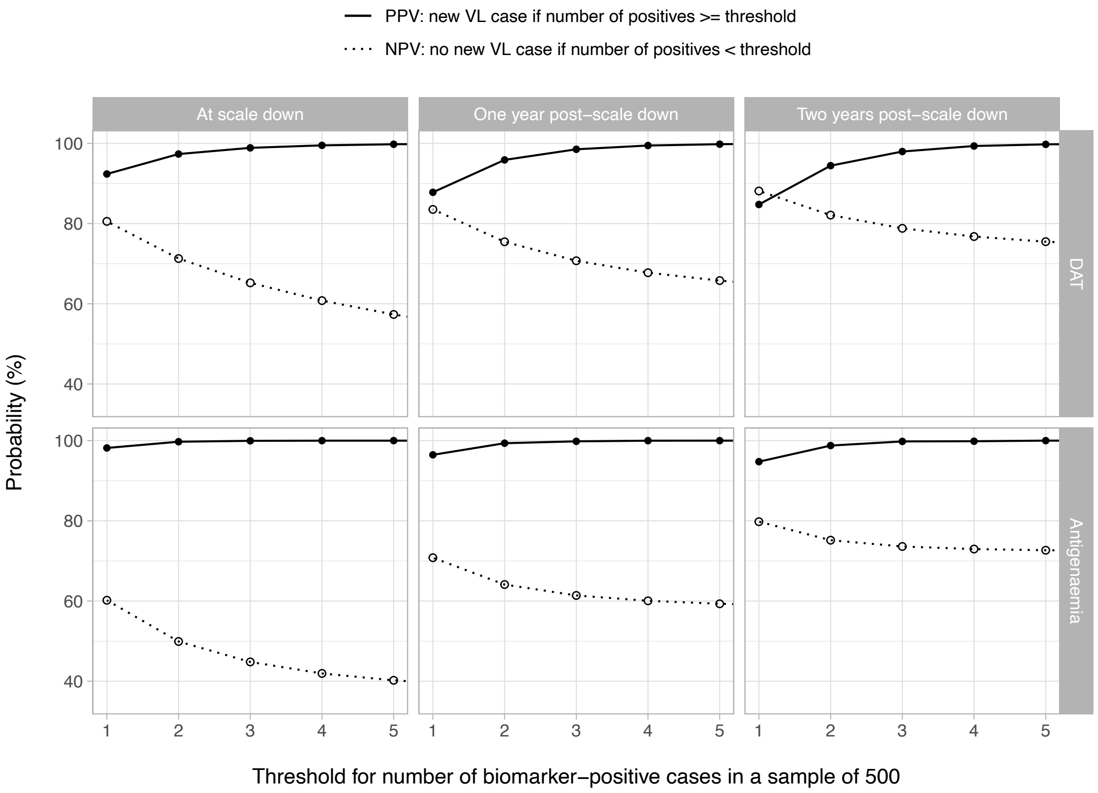

Figure S7. Positive and negative predictive value of DAT and antigenaemia prevalence in adults (age 15+) for occurrence of at least one new VL case, given a choice of threshold value. Columns depict curves based on biomarkers measured on three different time points; rows depict different biomarkers. Note that the predictive values based on biomarker prevalences measured one or two years after scale-down (middle and right panels) are conditional on no new VL cases having been detected since scale-down. Predictions are based on the assumptions that asymptomatic infections do not contribute to transmission (model E0) and that 500 individuals are tested for biomarker positivity.

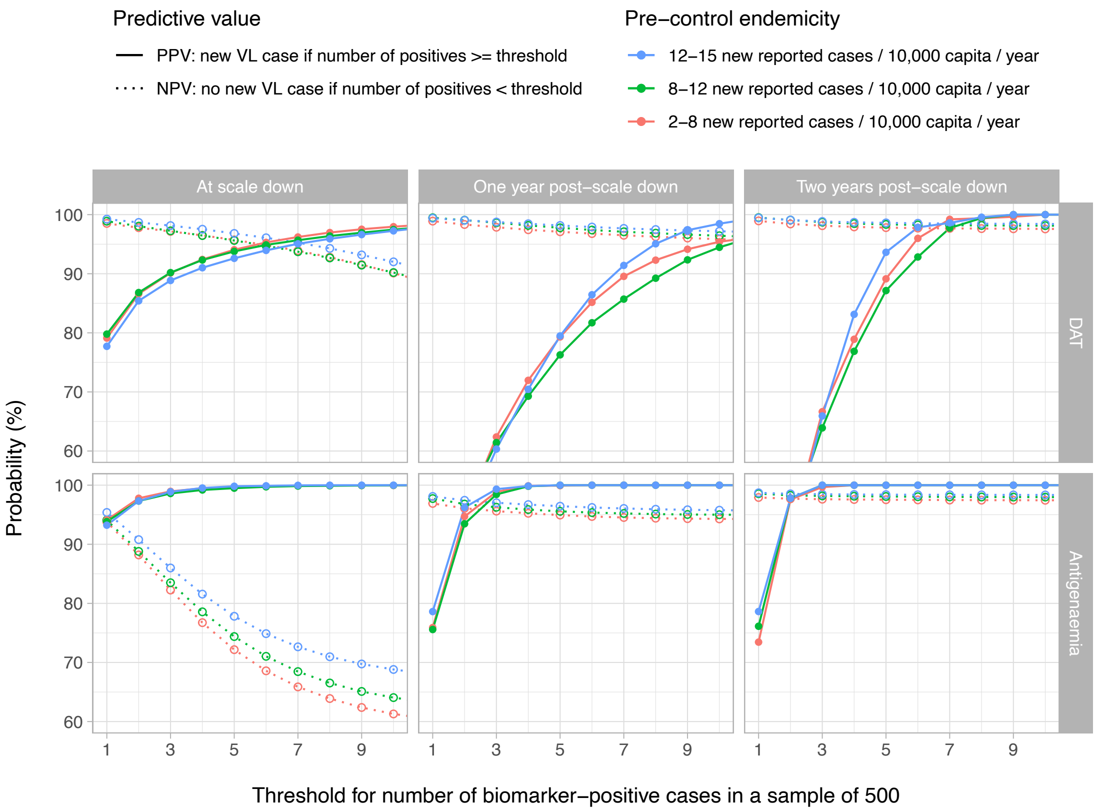

Figure S8. Positive and negative predictive value of DAT and antigenaemia prevalence in adults (age 15+) for occurrence of at least one new VL case, given a choice of threshold value. Colours represent tertiles of pre-control case incidence; the colour legend indicates incidences ranges in terms of cases per 10,000 population per year. Columns depict curves based on biomarkers measured on three different time points; rows depict different biomarkers. Note that the predictive values based on biomarker prevalences measured one or two years after scale-down (middle and right panels) are conditional on no new VL cases having been detected since scale-down. Predictions are based on the assumptions that both symptomatic and asymptomatic infections contribute to transmission (model E1) and that 500 individuals are tested for biomarker positivity.

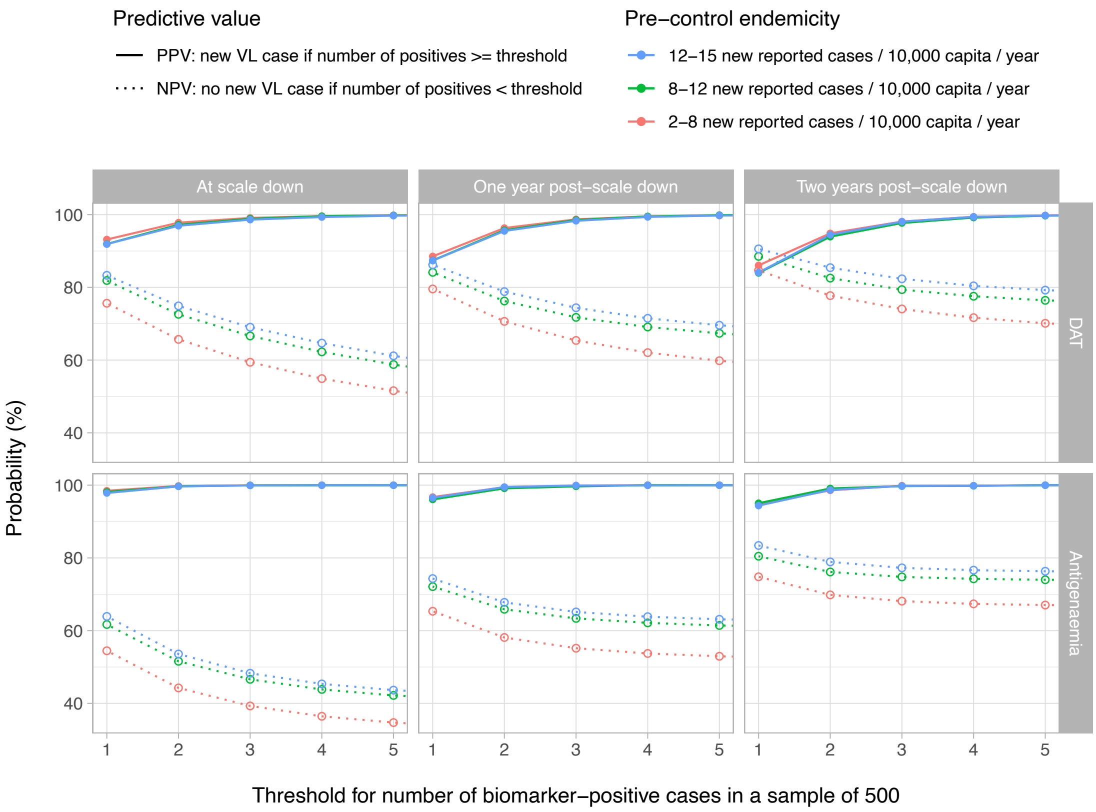

Figure S9. Positive and negative predictive value of DAT and antigenaemia prevalence in adults (age 15+) for occurrence of at least one new VL case, given a choice of threshold value. Colours represent tertiles of pre-control case incidence; the colour legend indicates incidences ranges in terms of cases per 10,000 population per year. Columns depict curves based on biomarkers measured on three different time points; rows depict different biomarkers. Note that the predictive values based on biomarker prevalences measured one or two years after scale-down (middle and right panels) are conditional on no new VL cases having been detected since scale-down. Predictions are based on the assumptions that asymptomatic infections do not contribute to transmission (model E0) and that 500 individuals are tested for biomarker positivity.

Table S3. Proportion of simulations with age-specific prevalence of DAT >0%, stratified by outcome (absence vs. occurrence of at least one new VL case) and time (years) since scaling down control efforts. Model E1 assumes that both symptomatic and asymptomatic individuals contribute to transmission, whereas in model E0, only cases of VL and PKDL transmit to sand flies.

		Model E0				Model E1			
New VL case(s) after scale-down	Time since scale-down	Age 0–4	Age 5–14	Age 15+	All ages	Age 0–4	Age 5–14	Age 15+	All ages
Yes	0	54.9	90.5	99.8	99.8	88.0	98.9	100.0	100.0
	1	64.3	92.1	99.8	99.8	91.9	99.1	99.9	100.0
	2	72.7	93.6	99.9	99.9	90.2	98.7	99.9	100.0
No	0	2.1	15.5	48.2	48.8	4.0	17.1	44.2	45.0
	1	1.2	10.5	35.0	36.1	2.0	11.1	34.0	34.8
	2	1.1	6.3	24.9	25.6	0.4	5.0	24.4	25.0

Supplementary Table S4. Proportion of simulations with age-specific prevalence of antigenaemia >0%, stratified by outcome (absence vs. occurrence of at least one new VL case) and time (years) since scaling down control efforts. Model E1 assumes that both symptomatic and asymptomatic individuals contribute to transmission, whereas in model E0, only cases of VL and PKDL can transmit to sand flies.

		Model E0				Model E1			
New VL case(s) after scale-down	Time since scale-down	Age 0–4	Age 5–14	Age 15+	All ages	Age 0–4	Age 5–14	Age 15+	All ages
Yes	0	24.2	71.3	95.7	96.2	56.8	94.2	98.8	98.8
	1	37.6	75.9	97.0	97.3	72.7	94.1	98.9	98.9
	2	49.0	81.5	97.4	97.6	75.2	91.5	98.2	98.2
No	0	0.3	2.8	15.2	15.9	0.9	5.3	12.9	13.4
	1	0.4	2.0	10.1	11.0	0.2	1.4	6.5	6.9
	2	0.5	1.5	6.7	7.3	0.0	0.3	2.2	2.4

References

1. World Bank. Crude birth rate per 1,000 population (India, 2011). http://data.worldbank.org/indicator, accessed 4 Sep 2015.
2. India Ministry of Health and Family Welfare. Health and Family Welfare Statistics in India 2013. https://data.gov.in/catalog/estimated-age-specific-death-rates-sex#web_catalog_tabs_block_10, accessed 4 Sep 2015.
3. Le Rutte EA, Coffeng LE, Bontje DM, et al. Feasibility of eliminating visceral leishmaniasis from the Indian subcontinent: explorations with a set of deterministic age-structured transmission models. Parasit Vectors **2016**; 9:24.
4. Le Rutte EA, Chapman LAC, Coffeng LE, et al. Elimination of visceral leishmaniasis in the Indian subcontinent: a comparison of predictions from three transmission models. Epidemics **2017**; 18:67–80.
5. Jervis S, Chapman LAC, Dwivedi S, et al. Variations in visceral leishmaniasis burden, mortality and the pathway to care within Bihar, India. Parasit Vectors **2017**; 10:601.
6. Le Rutte EA, Chapman LAC, Coffeng LE, et al. Policy Recommendations From Transmission Modeling for the Elimination of Visceral Leishmaniasis in the Indian Subcontinent. Clin Infect Dis **2018**; 66:S301–S308.
7. Maintz EM, Hassan M, Huda MM, et al. Introducing single dose liposomal amphotericin B for the treatment of visceral leishmaniasis in rural Bangladesh: Feasibility and acceptance to patients and health staff. J Trop Med **2014**; 2014.
8. Sundar S, Sinha PK, Rai M, et al. Comparison of short-course multidrug treatment with standard therapy for visceral leishmaniasis in India: An open-label, non-inferiority, randomised controlled trial. Lancet **2011**; 377:477–486.
9. Rahman KM, Islam S, Rahman MW, et al. Increasing Incidence of Post–Kala-Azar Dermal Leishmaniasis in a Population-Based Study in Bangladesh. Clin Infect Dis **2010**; 50:73–76.
10. Ramesh V, Kaushal H, Mishra AK, Singh R, Salotra P. Clinico-epidemiological analysis of Post kala-azar dermal leishmaniasis (PKDL) cases in India over last two decades: a hospital based retrospective study. BMC Public Health **2015**; 15:1092.
11. Uranw S, Ostyn B, Rijal A, et al. Post-Kala-azar Dermal Leishmaniasis in Nepal: A Retrospective Cohort Study (2000–2010). PLoS Negl Trop Dis **2011**; 5:e1433.
12. Le Rutte EA, Coffeng LE, Bontje DM, et al. Feasibility of eliminating visceral leishmaniasis from the Indian subcontinent: Explorations with a set of deterministic age-structured transmission models Quantitative analysis of strategies to achieve the 2020 goals for neglected tropical diseases: Wher. Parasites and Vectors **2016**; 9.
13. Mondal D, Bern C, Ghosh D, et al. Quantifying the Infectiousness of Post-Kala-Azar Dermal Leishmaniasis Toward Sand Flies. Clin Infect Dis **2019**; 69:251–258.
14. Le Rutte EA, Zijlstra EE, de Vlas SJ. Post-Kala-Azar Dermal Leishmaniasis as a Reservoir for Visceral Leishmaniasis Transmission. Trends Parasitol **2019**; 35:590–592.

15. Stauch A, Sarkar RR, Picado A, et al. Visceral Leishmaniasis in the Indian Subcontinent: Modelling Epidemiology and Control. PLoS Negl Trop Dis **2011**; 5:e1405.
16. Singh RP, Picado A, Alam S, et al. Post-kala-azar dermal leishmaniasis in visceral leishmaniasis-endemic communities in Bihar, India. Trop Med Int Heal **2012**; 17:1345–1348.
17. Desjeux P, Ghosh RS, Dhalaria P, Strub-Wourgaft N, Zijlstra EE. Report of the Post Kala-azar Dermal Leishmaniasis (PKDL) Consortium Meeting, New Delhi, India, 27-29 June 2012. Parasit Vectors **2013**; 6:196.
18. Kirk R, Lewis DJ. Studies in leishmaniasis in the Anglo-Egyptian Sudan. XI. Phlebotomus in relation to leishmaniasis in the Sudan. Trans R Soc Trop Med Hyg **1955**; 49:229–40.
19. Palit A, Bhattacharya SK, Kundu S. Gonotrophic cycle and age gradation of Phlebotomus argentipes in West Bengal , India. **2011**;
20. Sacks DL, Perkins PV. Development of infective stage Leishmania promastigotes within phlebotomine sand flies. Am J Trop Med Hyg **1985**; 34:456–459.
21. Hati A, Sur S, De N, et al. Longitudinal study on distribution of Phlebotomus argentipes sandflies at different heights in cattleshed. Indian J Med Res **1991**; 93:388–390.
22. Hati. AK, Palit A, Chakraborty S, S. Bhattacharya KKG and, S.Das. PHLEBOTOMUS ARGENTIPES ANNANDALE AND BRUNETTI (DIPTERA) CAUGHT ON MAN AT NIGHT IN A CLEAN BIOTOPE. Rec, zool, Surv India **1984**; 81:9–12.
23. Behrend MR, Basáñez M-G, Hamley JID, et al. Modelling for policy: The five principles of the Neglected Tropical Diseases Modelling Consortium. PLoS Negl Trop Dis **2020**; 14:e0008033.
